# Spontaneous Post-Orthopedic HIT-Like Syndrome: A Case Report and Literature Review with a Diagnostic and Therapeutic Guidance

**DOI:** 10.1055/a-2978-5120

**Published:** 2026-10-28

**Authors:** Maxime G. Zermatten, Noemy Friedrich, Francesco Grandoni, Mathilde Gavillet, Alessandro Aliotta, Lorenzo Alberio

**Affiliations:** 1 30635 Service and Central Laboratory of Hematology, Lausanne University HospitalLausanneSwitzerland

**Keywords:** heparin-induced thrombocytopenia, spontaneous HIT-like syndrome, delayed-onset HIT, case report

## Abstract

Spontaneous heparin-induced thrombocytopenia (spHIT)-like syndrome is a severe thromboembolic complication, most often occurring after total knee arthroplasty. We report an original case and review 25 published cases to propose diagnostic and therapeutic guidance for this challenging condition. A 57-year-old man developed thrombocytopenia and multiple thrombotic events, including pulmonary embolism, hepatic and renal vein thromboses, as well as bilateral adrenal hemorrhages, starting on postoperative day 7. Despite limited heparin exposure, anti-platelet factor 4 (PF4)/heparin antibodies and positive functional assays confirmed spHIT. Clinical deterioration occurred under heparin therapy, with subsequent ischemic stroke. Treatment with intravenous immunoglobulin (IVIG) and non-heparin anticoagulation (argatroban) led to rapid platelet recovery and favorable outcome. Based on this case and literature review, spHIT probability can be assessed using the 4T score, considering surgery day as day 0. Adrenal infarction or hemorrhage after orthopedic surgery should prompt suspicion of spHIT. Early initiation of non-heparin anticoagulation and high-dose IVIG is mandatory.

## Introduction

Spontaneous heparin-induced thrombocytopenia (spHIT)-like syndrome, a subtype of autoimmune heparin-induced thrombocytopenia (aHIT), is a rare but severe thromboembolic disorder that typically occurs after orthopedic surgery, particularly total knee arthroplasty (TKA). 1 2 aHIT is characterized by the presence of both heparin-dependent and heparin-independent anti-platelet factor 4 (PF4) antibodies capable of activating platelets, leading to thrombocytopenia and life-threatening thromboembolic complications. 1

aHIT encompasses several clinical subtypes, including spHIT and delayed-onset HIT (doHIT), the latter occurring after discontinuation of heparin therapy. 1 Distinguishing spHIT from doHIT may be challenging, particularly in patients with recent or minimal heparin exposure. Accurate identification of spHIT is clinically important, given its potential for more severe thrombotic complications and the need for more aggressive treatment compared to doHIT.

To date, 25 cases of spHIT have been published, with Warkentin and Greinacher additionally reporting seven unpublished cases. 1 Given its rarity, evidence-based guidelines for diagnosis and management are lacking.

In this report, we describe a new case of spHIT following TKA and propose practical diagnostic and therapeutic considerations for patients with severe thromboembolic manifestations in this clinical context.

## Case Report

A 57-year-old man was admitted to a peripheral hospital with epigastric pain on postoperative day (POD) 7 following TKA. Postoperative thromboprophylaxis consisted of enoxaparin 40 mg once daily from POD0 until POD3, followed by rivaroxaban 10 mg once daily thereafter ( Fig. 1 ).

**Fig. 1:**
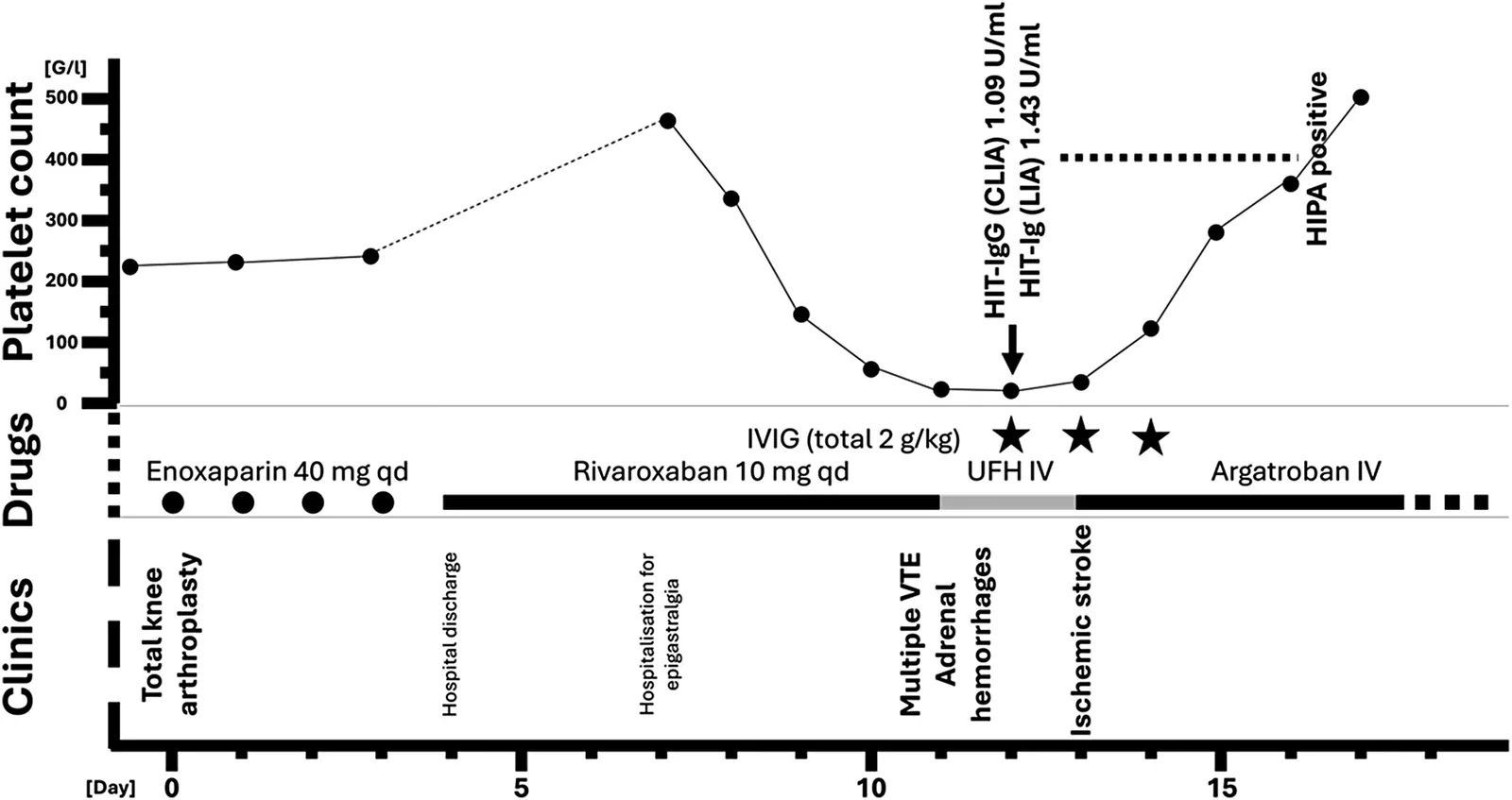
Clinical course of an unpublished case of spontaneous HIT-like syndrome (platelet count × 10 ^9^ /L). Stars represent the IVIG administrations (total 2 g/kg). CLIA, chemiluminescence immunoassay; HIPA, heparin-induced platelet aggregation assay; HIT, heparin-induced thrombocytopenia; IV, intravenous; IVIG, intravenous immunoglobulin; LIA, latex immunoassay; PE, pulmonary embolism; qd, once a day; UFH, unfractionated heparin; VTE, venous thromboembolic event.

At presentation on POD7, the platelet count was 473 × 10 ^9^ /L. A computed tomography (CT) scan revealed pneumonia, which was treated with piperacillin–tazobactam. From POD8 onwards, the patient experienced a rapid and profound decline in platelet count, reaching 38 × 10 ^9^ /L by POD11. On POD11, the patient developed hemodynamic instability, prompting further imaging, which demonstrated pulmonary embolism, hepatic and renal vein thromboses, as well as bilateral adrenal hemorrhage. Because enoxaparin had been administered for only 4 days ( Fig. 1 ), heparin-induced thrombocytopenia (HIT) was not initially suspected, and therapeutic anticoagulation with unfractionated heparin was initiated.

On POD12, the hematology consultant considered spHIT and required testing for anti-PF4/heparin antibodies in our reference laboratory. Because of shipping delay, the sample was tested on POD13. On POD12, inconsistently with the working hypothesis, but based on the “uncertain and non-quantified” pretest probability of spHIT, in the absence of anti-PF4/heparin antibody results, and because of the unavailability of alternative anticoagulants in the primary care center, unfractionated heparin was continued and low-dose intravenous immunoglobulin (IVIG; 0.4 g/kg) was administered. On POD13, anti-PF4/heparin antibody testing turned out positive (chemiluminescent immunoassay 1.09 U/mL and latex immunoassay 1.43 U/mL, interpreted according to the Lausanne Algorithm 3 ) and the patient suffered an acute ischemic stroke involving the superficial territory of the anterior cerebral artery, accompanied by acute confusion. Unfractionated heparin was discontinued, a second IVIG dose (1 g/kg) was administered, and the patient was transferred to our tertiary care center for treatment with argatroban. IVIG dose was completed on POD14 (0.6 g/kg) to reach a total dose of 2 g/kg.

The clinical course was favorable, with platelet count recovery >150 × 10 ^9^ /L already by POD14. A positive heparin-induced platelet aggregation (HIPA) assay confirmed the diagnosis of spHIT, demonstrating heparin-dependent platelet activation in three out of four tested donor platelet samples (performed at Universitätsmedizin Greifswald). Given the brief exposure to enoxaparin, doHIT is less likely but could not be completely excluded (see “Discussion” section).

### Differential Diagnostic Considerations

Several differential diagnoses were considered in view of the severe and multisystem thromboembolic manifestations. Catastrophic antiphospholipid syndrome (CAPS) was initially suspected due to involvement of more than three organ systems. However, antiphospholipid antibodies were negative on chemiluminescent assays (anticardiolipin IgG 14.7 CU [99th percentile 35], IgM 5.8 CU [99th percentile 45]; anti-β2-glycoprotein I IgG 8.8 CU [99th percentile 18], IgM < 1.1 CU [99th percentile 10]), effectively excluding CAPS. Trousseau syndrome was also ruled out based on the absence of malignancy on extensive imaging, including cerebral angio-CT and magnetic resonance imaging (MRI), as well as cervical, thoracic, abdominal, and pelvic CT scans. In addition, paradoxical embolism as a cause of stroke was excluded in the absence of a *patent foramen ovale* or other shunts on microbubble contrast echocardiography.

## Literature Review

Details of our patients and the 25 previously published cases of spHIT are summarized in Table 1 . 1 4 5 6 7 8 9 10 11 12 13 14 15 16 17 18 19 20 21 22 23

**Table 1:** Overview of the published post-orthopedic spontaneous heparin-induced thrombocytopenia cases.

	Publication	Age (years)	Surgery	Timing of thrombocytopenia (POD)	Timing of events (POD)	4T score	Thromboembolic events	Adrenal hemorrhage/thrombosis	Acute treatment	Further treatment	Outcome	Argatroban	Fondaparinux	IVIG	Argatroban resistance	Fondaparinux resistance
Cases originally reported as spHIT	**Synthesis**	**NA**	**92% total knee arthroplasty**	**8–28**	**7–28**	**≥5**	**100%, often severe manifestations**	**52% (13/** **25)**	**Alternative anticoagulation IVIG**	**VKA/DOAC (variable duration)**	**Mortality 4% (1/** **25)**	**76% (16/** **21)**	**19% (4/** **21)**	**29% (6/** **21)**	**38% (6/** **16)**	**50% (2/** **4)**
	Illustrative case, 2026	57	Total knee arthroplasty	8	11, 13	7	Renal vein, hepatic vein thromboses, PE; CVI under heparin	Yes	Argatroban + IVIG (total 2 g/kg on 3 days)	Acenocoumarol (3 months)	Recovery	Yes	No	Primary	No	NA
	Langer et al, 2026 23	60	Total hip arthroplasty	34	34	6	PE, bilateral TVP	No	Bivalirudin + IVIG (total 2 g/kg on 2 days)	Apixaban	Recovery	No	No	Yes	NA	NA
	Korolyov and Hu, 2025 21	83	Total knee arthroplasty	NR	NR	6	Lower extremity distal DVT	No	Argatroban, fondaparinux, dexamethasone	Apixaban (unclear duration)	Recovery	Yes	Yes	No	No	No
	Olevsky and Rosove, 2021 4	57	Total knee arthroplasty	10	10	7	Segmental PE, acute left popliteal DVT	No	Argatroban	Rivaroxaban (6 months)	Recovery	Yes	No	No	No	NA
	Olevsky and Rosove, 2021	70	Total knee arthroplasty	NR	NR	6	Femoral artery thrombosis, with re-thrombosis and amputation/PE under UFH	No	Argatroban	Rivaroxaban (unclear duration)	Recovery, amputation	Yes	No	No	No	NA
	Warkentin and Greinacher, 2021 1	70	Total knee arthroplasty	Before 28	28, 36, 38	7	Popliteal DVT; iliac artery thrombosis; re-thrombosis (DVT, PE, arterial), amputation	No	Argatroban with thromboses, rivaroxaban 2× 15 mg, IVIG	Rivaroxaban (2× 15 mg)	Partial recovery, amputation	Yes	No	For resistance	Yes	NA
	Saengboon et al, 2021 8	68	Bilateral total knee arthroplasty	12	12, 16, 18	6	Lower extremity arterial occlusion, progression under UFH	Yes	Fondaparinux IVIG (1 g/kg on 2 following days)	Apixaban (6 months)	Recovery	No	Yes	Primary	NA	No
	Hwang et al, 2020	56	Total knee arthroplasty	11	11	7	Cerebral venous sinus thrombosis, DVT of the left internal jugular vein	No	Bivalirudin IVIG (1 g/kg on 2 following days)	Long-term warfarin	Recovery	No	No	Primary	NA	NA
	Swarup et al, 2020 10	33	Left femoral and neck curettage, bone grafting	10	10	8	Central PE, extensive proximal and distal DVT	No	Argatroban	Warfarin (2 years)	Recovery	Yes	No	No	No	NA
	Hornick and Ayafor, 2020 20	51	Bilateral total knee arthroplasty	21	16	6	Bilateral distal central PE, bilateral femoral and popliteal DVT	No	Rivaroxaban	Rivaroxaban (6 months)	Recovery	No	No	No	NA	NA
	VanderVeer et al, 2019 11	68	Total knee arthroplasty	9		8	Only adrenal hemorrhage	Yes	Prophylactic rivaroxaban until day 28	NA	Recovery	No	No	No	NA	NA
	Alidoost et al, 2019 12	68	Total knee arthroplasty	12	8	7	Small PE; after IVC filter placement: DVT from the IVC to the lower extremity	Yes	Argatroban	Warfarin (unclear duration)	Recovery	Yes	No	No	No	NA
	Mohanty et al, 2019 13	52	Total knee arthroplasty	12	12	8	Mesenteric ischemia and mesenteric vein thrombosis	No	Argatroban/fondaparinux, IVIG (1 g/kg on 2 following days)	Fondaparinux (unclear duration)	Recovery	Yes	Yes	For resistance	Yes	Yes
	Poudel et al, 2017 14	53	Total knee arthroplasty	15	14	6	Acute PE, DIC	No	Fondaparinux with resistant DIC, argatroban with new DVT, rivaroxaban	Rivaroxaban (27 weeks)	Recovery	Yes	Yes	No	Yes	Yes
	Baker and Lim, 2017 15	72	Total knee arthroplasty	12	12	7	Bilateral PE, left cephalic, left lower extremity, right posterior tibial vein DVT	No	Argatroban, ICH indicating IVC filter	Apixaban (3 months)	Recovery, ICH	Yes	No	No	No	NA
	DeBaun et al, 2017 22	68	Bilateral total knee arthroplasty	13	13	5	Proximal lower extremity DVT	Yes	Argatroban	Apixaban (unclear duration)	Recovery	Yes	No	No	No	NA
	Elshoury et al, 2015 16	48	Total knee arthroplasty	8	9, 10	8	Bilateral PE	Yes	Argatroban with recurrent PE, IVC filter	Warfarin	Recovery	Yes	No	No	Yes	NA
	Warkentin et al, 2015 5	71	Total knee arthroplasty	12	12, 13	7	PE, inferior vena cava and popliteal DVT	Yes	Argatroban	Fondaparinux (3 months)	Recovery	Yes	No	No	No	NA
	Warkentin et al, 2014 6	54	Shoulder hemiarthroplasty	15	15	7	Lower-extremity DVT; acute CVI of the left posterior inferior cerebellar artery territory	No	Argatroban	Fondaparinux (3 months)	Recovery	Yes	No	No	No	NA
	Ketha et al (four cases), 2013 17	60, 65, 71, 61	Total knee arthroplasty	NR	NR	5, 5, 6, 6	Only adrenal hemorrhage	Yes	NR	NR	NR	NR	NR	NR	NR	NR
	Mallik et al, 2011 18	60	Bilateral total knee arthroplasty	10	7	7	Left popliteal DVT, bilateral lower extremity DVT under argatroban	No	Argatroban, IVIG (1 g/kg on 2 following days) after re-thrombosis	Warfarin (10 months)	Recovery	Yes	No	For resistance	Yes	NA
	Pruthi et al, 2008	61	Total knee arthroplasty	9	8	8	Saphenous SVT, non-occlusive popliteal and femoral DVT	Yes	IVC filter, argatroban with PE and progression of DVT, lepirudin	Warfarin (unclear duration)	Recovery	Yes	No	No	Yes	NA
	Jay and Warkentin, 2008 7	69	Total knee arthroplasty	8	8	8	Lower extremity DVT; digital infarcts; DIC, death	Yes	Danaparoid	NR	Recovery, death from anoxic brain damage	No	No	No	NA	NA
Potential spHIT cases originally reported as other types of HIT	Alsaleh et al, 2008 29	64	Total knee arthroplasty (LMWH for 3 days)	Between 7 and 11	13	6	Proximal lower extremity DVT; cerebral ischemia under danaparoid	Yes	Danaparoid, argatroban	Warfarin (unclear duration)	Recovery	Yes	No	No	No	NA
	Salem et al, 2010 30	67	Total knee arthroplasty (fondaparinux)	9	9	7	Ischemic stroke, bilateral proximal lower extremity DVT	No	Argatroban	Warfarin	Recovery, vegetative state	Yes	No	No	No	NA
	Tardy-Poncet et al, 2015 28	71	Total knee arthroplasty (heparin in Cell Saver)	10	NA	5	No thromboembolic events, only thrombocytopenia	No	Dabigatran	Dabigatran	Recovery	No	No	No	NA	NA
	Bauman et al, 2022 31	57	Total knee arthroplasty (LMWH for 3 days)	11	11	5	Cerebral venous sinus thrombosis	No	Bivalirudin	Warfarin (6 months)	Recovery	No	No	No	NA	NA

Abbreviations: DIC, disseminated intravascular coagulation; DOAC, direct oral anticoagulant; DVT, deep vein thrombosis; IVC, inferior vena cava; IVIG, intravenous immunoglobulin; LMWH, low-molecular-weight heparin; NA, not applicable; NR, not reported; PE, pulmonary embolism; POD, postoperative day; spHIT, spontaneous heparin-induced thrombocytopenia; SVT, superficial vein thrombosis; UFH, unfractionated heparin; VKA, vitamin K antagonist.

Regarding diagnosis, 23 of 26 cases (88%) occurred after TKA. At the time of spHIT diagnosis, the 4T score, calculated with the day of surgery as day 0, ranged from 5 to 8.

With respect to clinical manifestations, all patients developed thromboembolic complications. Venous thromboembolic events occurred in 21 of 26 cases (80.8%), and arterial events in 7 of 26 cases (26.9%). Remarkably, adrenal hemorrhage occurred in 13 of 26 cases (50.0%). Given its disproportionately high incidence, adrenal hemorrhage secondary to adrenal vein thrombosis may represent a distinctive clinical manifestation of spHIT and was therefore assessed separately from other thrombotic events in Table 1 . One patient (3.8%) died because of thromboembolic complications.

Acute management was documented in 22 cases. Most patients received argatroban as initial non-heparin anticoagulant therapy (15/22 cases, 68.2%). One additional patient received argatroban as second-line because of fondaparinux resistance. Six of these patients (6/16, 37.5%) experienced new or progressive thrombotic events despite argatroban (argatroban resistance, possibly due to “aPTT confounding” impacting monitoring and leading to underdosing 1 24 ). Out of the 22 patients, 2 (9.1%) received fondaparinux initially, and 2 secondarily, 1 for argatroban resistance and 1 for a rash on argatroban; of these 4 patients, 2 failed to respond to fondaparinux (and argatroban) and were treated with IVIG (see below). Other immediate alternative anticoagulant strategies in single patients included therapeutic-dose bivalirudin, danaparoid, rivaroxaban, and prophylactic-dose rivaroxaban. Inferior vena cava filters were placed in four patients, with filter-associated thromboses reported in one case (25%). Subacute anticoagulation strategies varied widely due to lack of evidence, ranging from short-term prophylactic rivaroxaban for 28 days to long-term anticoagulation with warfarin.

Three patients ultimately received IVIG (total dose 2 g/kg), either prophylactically in addition to alternative anticoagulation or as add-on therapy after thrombotic event recurrence despite anticoagulation, with favorable outcomes reported.

IVIG may be particularly valuable in HIT and related diseases (aHIT and spHIT). Indeed, pathogenic immune complexes activate platelets through FcγRIIa. 25 High concentrations of inert IgG, as achieved with IVIG, may competitively interfere with this FcγRIIa-mediated pathway and thereby suppress platelet activation. This effect may be particularly relevant in heparin-independent platelet activation, supporting the use of IVIG in spHIT, aHIT, and vaccine-induced immune thrombotic thrombocytopenia (VITT). 26

## Discussion

Here, we report on a previously unpublished case of spHIT ( Fig. 1 ) and provide an overview of the 25 other published cases ( Table 1 ).

In the present case, in which the patient was exposed to four doses of enoxaparin 40 mg, a differential diagnosis of classic HIT and doHIT may be considered. Although cases of HIT following only very brief heparin exposure have been reported (including one case after a single unfractionated heparin exposure after hip surgery), 5 27 such a brief exposure (<5 days), particularly to low-molecular-weight heparin, is not considered sufficient to trigger classical HIT. Notably, three cases of doHIT and one case of fondaparinux-associated HIT after TKA have been reported. 28 29 30 31 These cases, especially the one of Alsaleh et al, closely resemble the one described here and demonstrate the difficulty in distinguishing spHIT from doHIT in clinical practice. These cases illustrate the diagnostic challenges encountered within the spectrum of PF4-related disorders, highlighting the close pathophysiological and clinical overlap among these entities.

Nevertheless, based on the literature review, the timing of symptom onset after TKA, the very brief exposure to low-molecular-weight heparin, and the severity of the thromboembolic manifestations, the present case is most consistent with a diagnosis of spHIT. This interpretation may also apply to the case reported by Alsaleh et al. 29

Among the 25 published cases of spHIT, four key observations emerge from our literature review. First, TKA appears to be by far the strongest predisposing factor. Second, thromboembolic manifestations are particularly severe and often multisystemic. They typically occur within 2 weeks following TKA but can arise up to 1 month after surgery. Third, adrenal hemorrhage is a frequent and characteristic complication, warranting rapid consideration of an underlying adrenal vein thrombosis as the primary cause of a secondary hemorrhage 32 and recognition of spHIT in the appropriate clinical context. Fourth, resistance to alternative anticoagulation (especially argatroban and fondaparinux) is relatively common, highlighting the extremely severe prothrombotic state associated with spHIT, which cannot be controlled with standard-dose anticoagulation. Early administration of high-dose IVIG, saturating the FcγRIIa, appears to be necessary for downregulating the underlying mechanism.

Based on these findings, we propose the following diagnostic and therapeutic approach for suspected spHIT.

### Diagnostic Approach

- Consider spHIT in patients who develop thrombocytopenia and/or thromboembolic events 5–34 days after TKA.
- Estimate pretest probability using the 4T score, considering the day of surgery as day 0 (instead of the day on which heparin administration began).
- In cases of intermediate or high pretest probability, promptly perform HIT immunoassays and functional platelet activation assays.

### Therapeutic Approach in Patients with Confirmed spHIT, or in Whom spHIT is Strongly Suspected and Cannot be Rapidly Excluded

- Immediately switch anticoagulation to a therapeutic-dose non-heparin anticoagulant.
- Administer high-dose IVIG (1 g/kg daily for 2 days, total 2 g/kg).

In conclusion, spHIT is a rare but life-threatening complication following orthopedic surgery, particularly TKA. Despite its rarity, clinicians should maintain a high index of suspicion, especially in the presence of adrenal hemorrhage (>50% of spHIT), as delayed recognition and lack of adequate treatment are associated with substantial morbidity and mortality. Although diagnostic and therapeutic management is not supported by high-level evidence, we propose pragmatic guidance to assist clinicians in the management of this severe condition.

## Declaration of GenAI Use

During the preparation of this manuscript, the authors used assisted technologies: ChatGPT (OpenAI, GPT-5.5), solely to assist with editing statement: author-written text by improving clarity and readability and reducing the word count. The AI tool was not used to generate scientific content, analyze data, interpret results, or draw conclusions. All scientific content was written and reviewed, by the authors, who take full responsibility for the final manuscript.
